# Evaluating Epidemiology and Improving Surveillance of Infections Associated with Health Care, United States

**DOI:** 10.3201/eid2109.150508

**Published:** 2015-09

**Authors:** Shelley S. Magill, Ghinwa Dumyati, Susan M. Ray, Scott K. Fridkin

**Affiliations:** Centers for Disease Control and Prevention, Atlanta, Georgia, USA (S.S. Magill, S.K. Fridkin);; University of Rochester Medical Center, Rochester, New York, USA (G. Dumyati);; New York Emerging Infections Program, Rochester (G. Dumyati);; Emory University School of Medicine, Atlanta (S. Ray);; Georgia Emerging Infections Program, Decatur, Georgia, USA (S. Ray)

**Keywords:** epidemiology, nosocomial infections, antimicrobial resistance, health care, surveillance, United States, MRSA, methicillin-resistant Staphylococcus aureus, bacteria, enteric infections, Clostridium difficile, Candida, candidemia, multidrug-resistant gram-negative bacilli, Emerging Infections Program, EIP

## Abstract

The Healthcare-Associated Infections Community Interface (HAIC), launched in 2009, is the newest major activity of the Emerging Infections Program. The HAIC activity addresses population- and laboratory-based surveillance for *Clostridium difficile* infections, candidemia, and multidrug-resistant gram-negative bacilli. Other activities include special projects: the multistate Healthcare-Associated Infections and Antimicrobial Use Prevalence Survey and projects that evaluate new approaches for improving surveillance. The HAIC activity has provided information about the epidemiology and adverse health outcomes of health care–associated infections and antimicrobial drug use in the United States and informs efforts to improve patient safety through prevention of these infections.

## Abstract 2

This national resource provides much-needed data on pathogens, infections, and antimicrobial drug use.

Health care–associated infections (HAIs) and inappropriate antimicrobial drug use are major threats to patient safety in US health care facilities. For several years, the elimination of infections associated with health care has been a priority of the US Department of Health and Human Services and a “winnable battle” for the Centers for Disease Control and Prevention (CDC) (*1*). Essential to the development and implementation of effective HAI prevention and antimicrobial stewardship policies and practices is a current and comprehensive understanding of the epidemiology of HAIs and drug-resistant pathogens that commonly cause such infections.

The Emerging Infections Program (EIP) network, a CDC-supported, public health surveillance and research network, has conducted population-based surveillance for severe bacterial infections since 1995 through the Active Bacterial Core surveillance (ABCs). This program has successfully characterized the magnitude of infections, the patient populations affected, and risk factors for infections. Until 2004–2005, when the ABCs initiated surveillance for invasive methicillin-resistant *Staphylococcus aureus* (MRSA) infections, pathogens tracked by EIP were primarily associated with communities rather than with health care. In 2005, CDC’s National Nosocomial Infections Surveillance System, a longstanding, hospital-based surveillance system for HAIs, was integrated into the new National Healthcare Safety Network (NHSN). With the rapid expansion of NHSN during 2006–2010, additional complementary approaches were needed to define more fully the epidemiology of HAIs, drug-resistant pathogens, and antimicrobial drug use in US health care settings. Consequently, the Healthcare-Associated Infections Community Interface (HAIC) activity was launched to address this need; to bring together existing EIP HAI-related work into a single organizational structure (except for invasive methicillin-resistant *Staphylococcus aureus* surveillance, which remained part of the ABCs); and to develop further the EIP’s involvement and expertise in HAI epidemiology. The HAIC activity was initiated because of a growing need for a flexible infrastructure in which to conduct HAI-related surveillance and applied research activities and because of the increasing role of state health departments in the implementation of reporting and preventing HAIs through regional and statewide collaboration.

Over the past 5 years, the HAIC activity has become a national public health resource for data on urgent and emerging infectious diseases related to health care. The HAIC activity seeks to promote patient safety and health care quality through 2 main initiatives: 1) evaluation of the epidemiology and public health effects of HAIs to understand emerging pathogens and populations at risk; and 2) exploration of innovations to improve national surveillance and evaluation of HAI prevention and control strategies.

## Current HAIC Activities and Methods

HAIC activity projects are divided into 2 major categories: 1) pathogen-specific, population- and laboratory-based surveillance (for which 2 projects predated the formation of the HAIC activity); and 2) epidemiologic innovations. The HAIC activity currently conducts population-based surveillance aimed at defining the effects of disease and the epidemiology of infections caused by *Clostridium difficile*, *Candida* species (bloodstream infections only), and carbapenem-resistant *Enterobacteriaceae* (CRE) and *Acinetobacter baumannii* cultured from urine and sterile body sites (*2*). Although each of these 3 surveillance projects has its own case definition, catchment area, and data collection, all use laboratory-based criteria to identify cases. In addition, all 3 projects collect and submit isolates to CDC for further characterization, and they all collect data from medical records to confirm patient eligibility as a case, obtain demographics, and classify cases as either community associated or health care associated. When disease burden is high and surveillance catchment areas are large, CDC can work with specific EIP sites to develop medical records reviews and isolate sampling strategies that reduce resources needed for surveillance.

In 2008, population-based candidemia surveillance began in 2 EIP sites (Georgia and Maryland) to follow up previous surveillance conducted in the 8-county metropolitan area of Atlanta, Georgia, and in San Francisco, California, during 1992–1993 (*3*) and in Baltimore City and Baltimore County, Maryland, and in Connecticut during 1998–2000 (*4*). The primary objective of the ongoing surveillance is to assess changes in the incidence and epidemiology of these infections, including changes in antifungal resistance. Cases are identified through blood cultures that are positive for *Candida* species in residents of catchment areas. Submission and study of isolates enables a better understanding of antifungal susceptibility patterns among invasive *Candida* isolates; this information is not usually available from hospital clinical microbiology laboratories. Analysis of data collected during 2008–2011 in Georgia and Maryland showed marked declines in candidemia in infants, the group that had the highest rates of infection in the 1990s (*5*). The data also showed relatively stable levels of fluconazole resistance among *Candida* bloodstream isolates (*6*). Subsequent analyses identified increases in echinocandin-resistant and multidrug-resistant *Candida* infections during 2008–2012 (*7*). After sites in Oregon and Tennessee were added in 2011, candidemia surveillance is now conducted in 4 EIP sites, covering a population of 7.7 million persons. Data from this expanded surveillance are used to describe candidemia in these populations and to evaluate the emergence of echinocandin resistance in *C. glabrata* (*8*).

Surveillance for *C. difficile* infections (CDIs) began in 2009 and expanded by 2011 to include all of the 10 EIP sites and a population of ≈11.5 million persons. The objectives of CDI surveillance are to compile national estimates for CDIs associated with the community and with health care, to describe the epidemiology of these CDIs, and to characterize *C. difficile* strains. CDI surveillance captures the broad spectrum of CDI cases that occur in all community and health care settings (including nursing homes and facilities for rehabilitation and acute care) and collects extensive clinical and microbiologic data. CDI cases are defined on the basis of *C. difficile*–positive toxin or molecular assays for catchment area residents >1 year of age. Clinical data are used to confirm that patients had symptoms consistent with CDI, and epidemiologic data are used to classify cases into 1 of 3 categories: community associated; community-onset, health care facility associated; and health care facility onset. *C. difficile* isolates are collected from a convenience sample of laboratories and sent to CDC for molecular characterization, which enables comparative analysis of disease characteristics by strain type. Outcome data such as recurrence, hospitalization, and death are also captured.

This surveillance project has contributed substantially to the current understanding of CDI epidemiology in the United States. A recently published analysis of CDI surveillance data estimated that ≈453,000 CDI cases and 29,000 deaths occurred among patients with CDI in the United States in 2011 (*9*). Data from this surveillance project have also been used to evaluate differences in CDI incidence across EIP sites and have illustrated the importance of adjusting for patient factors (e.g., age, gender, and race) and hospital factors (e.g., inpatient days and use of nucleic acid amplification tests [NAAT]) for comparisons among populations (*10*). Data from EIP CDI surveillance have also shown substantial increases in CDI detection because laboratories have adopted NAAT for CDI diagnosis (*11*). EIP surveillance data have also enabled additional advances in the characterization of CDI: identification of outpatient health care exposures (e.g., doctor or dentist visits) among patients with community-associated CDI (*12*); description of the epidemiology of CDI in children, in whom most disease is community associated (*13*); evidence of the association between the North American pulsed-field gel electrophoresis type 1 epidemic *C. difficile* strain and more severe CDI outcomes (*14*); and description of the association between adoption of NAAT by clinical laboratories and implementation of stricter criteria for submitting stool specimens for testing (*15*). The CDI surveillance data are also used to estimate potential effects of reducing antimicrobial drug use on CDI rates (*16*), to estimate the incidence and outcome of CDI infection in nursing home populations (*17*), and to evaluate risk factors for community-associated infection. Ongoing surveillance will also enable measurement of outcomes of prevention efforts associated with inpatient antimicrobial drug stewardship or, potentially, with a CDI vaccine.

The third HAIC activity surveillance project targets multidrug-resistant gram-negative bacilli (MDR GNB). This project, known as the Multisite Gram-Negative Bacilli Surveillance Initiative (MuGSI), began in Georgia and Minnesota in 2010 as pilot projects and expanded to Oregon in 2011. The impetus for initiating population-based EIP surveillance for MDR GNB was the emergence of CRE in the United States. Patients infected with these organisms have few or sometimes no antimicrobial drug treatment options. The incidence and characteristics of MDR GNB are in flux, so a flexible yet specific surveillance program is needed. The program must be able to adapt to changing laboratory breakpoints and case definitions when needed to better define the impact of these infections, determine the populations at risk, and inform prevention efforts.

The main objective of MuGSI is to describe the epidemiology and population-based incidence of carbapenem-nonsusceptible *Enterobacteriaceae* species and *Acinetobacter baumannii*. The project also seeks to characterize isolates and describe resistance mechanisms among a subset of carbapenem-nonsusceptible *Enterobacteriaceae* isolates submitted to CDC. This surveillance has expanded in recent years to cover a surveillance area of ≈15 million persons in 8 states: Georgia, Oregon, Minnesota, Colorado, Maryland, New Mexico, New York and Tennessee. Initially, cases were defined by carbapenem-nonsusceptible (excluding ertapenem) and extended-spectrum cephalosporin-resistant *Escherichia coli*, *Enterobacter aerogenes*, and *E. cloacae*, *Klebsiella pneumoniae* and *K. oxytoca,* and carbapenem-nonsusceptible (excluding ertapenem) *Acinetobacter baumannii* complex isolated from normally sterile sites or from urine of residents in the surveillance areas. In MuGSI surveillance, most cases are identified through queries of automated susceptibility-testing instruments in clinical laboratories that serve the catchment areas rather than through routine output of summarized test results (often called line listings) generated by laboratory information systems (*18*). This method enables the application of case definitions based on antimicrobial drug susceptibility test results that may be suppressed from routine reports entered into the patient’s medical record. Also, depending on the concentration range of drugs tested, the method enables application of the latest breakpoints from the Clinical Laboratory Standards Institute (http://www.clsi.org/) before they have been widely implemented by clinical laboratories. Isolates from EIP sites are being used to evaluate different phenotypic definitions used to identify carbapenemase-producing CRE (*19*). Data from this evaluation have assisted in modifying CRE definitions used for reporting to NHSN and for updating the MuGSI case definition. Finally, MuGSI is uniquely positioned to describe persons with community-associated CRE.

Since its inception, the HAIC activity has also conducted several projects in epidemiology innovations, a major area of growth for the HAIC activity. The largest of these projects is a multicenter HAI and antimicrobial drug use prevalence survey project. This multiphase effort is designed to fill gaps in data collected through NHSN by developing and conducting a national-scale point prevalence survey that estimates the scope and magnitude of all HAIs affecting acute-care hospital patients. This project also describes the nature of and rationale for antimicrobial use in acute care hospitals. The project development began in 2009 with a single-city pilot survey (*20*). A limited roll-out survey was conducted in 22 hospitals in the 10 EIP sites in 2010, followed by the full-scale survey in 183 hospitals across the 10 sites in 2011. Data from the full-scale survey were used to establish the current annual estimates of HAIs in US acute-care hospitals: ≈722,000 infections in 648,000 patients (*21*). The survey showed that surgical site infections and pneumonias were the most common HAIs and also that device-associated infections, which have for many years been the focus of most HAI prevention efforts, accounted for only 26% of all HAIs. *C. difficile* was the most common pathogen causing HAIs; considering the importance of antimicrobial drug use in the epidemiology of CDI, this finding supports CDC’s increasing focus on antimicrobial stewardship programs in acute-care hospitals. The antimicrobial drug use component of the survey showed that half of all patients included in the survey were receiving antimicrobial drugs at the time of the survey; furthermore, broad-spectrum antimicrobial drug use was very common, even among patients with community-onset infections and among patients who were not in critical care units (*22*). 

The next phase of the prevalence survey, scheduled for 2015, includes a hospital infection control and antimicrobial stewardship practices questionnaire; it also has assessments of the quality of antimicrobial drug prescribing for selected clinical scenarios. The prevalence survey is an effective approach for obtaining broad, situational awareness of HAIs and antimicrobial drug use in different health care settings, particularly those settings where robust, prospective surveillance is not yet available or widely used. These surveys have been used in many other countries, including a European Union survey conducted in 2011–2012 (*23*). The methods for the US survey effort were developed with input from European colleagues, including those in the European Centre for Disease Prevention and Control, in an attempt to enable comparative metrics. We also relied on European colleagues’ considerable experience in conducting HAI and antimicrobial use prevalence surveys in long-term care facilities (*24*). We consulted them for input on a pilot EIP HAIC antimicrobial drug use prevalence survey for nursing home HAIs. This pilot was conducted in 9 nursing homes in 4 EIP sites, and expansion to a larger-scale, US nursing home survey in the future is being considered.

Other innovations projects have sought to field-test streamlined, simplified methods for conducting HAI surveillance in NHSN. One example of these short-term innovations projects is a device-associated HAI denominator data simplification project to identify streamlined sampling methods that can replace daily collection of patient- and device-day data (*25**,**26*). Other innovations projects include field-testing of a new surveillance component for CDI and urinary tract infections (UTIs) in long-term care facilities and field-testing of a definition modification of bloodstream infections associated with central lines (*27*). Another innovations project is surveillance for bloodstream infections in dialysis facilities (*28*). EIP sites have also completed work to validate data submitted to NHSN. For example, the Connecticut and New Mexico EIP sites have compared MRSA bacteremia and CDI data collected through EIP’s population-based surveillance with MRSA and CDI Laboratory-Identified Event data (http://www.cdc.gov/nhsn/labid-calculator/index.html) submitted to NHSN (*29*). Knowledge gained through these EIP projects has directly affected several NHSN surveillance operations: in 2015, implementation of surveillance of central line–associated bloodstream infection and catheter-associated urinary tract infection denominator sampling methods (http://www.cdc.gov/nhsn/acute-care-hospital/clabsi/index.html); also in 2015, the addition of selected variables to Laboratory-Identified Event reporting to improve the completeness of case information capturing; in 2014, implementation of the Mucosal Barrier Injury-Laboratory Confirmed Bloodstream Infection definition; and in 2013, clarification of UTI surveillance methods for long-term care facilities.

Data from HAIC activity population-based surveillance projects and from the HAI and antimicrobial drug use prevalence survey have been critical to the development of recent high-profile reports. Of the 18 pathogens or pathogen groups included as serious, urgent, or concerning threats to public health in CDC’s first report on antimicrobial threats in the United States, discussions of 7 used estimates from HAIC activity data (*30*). HAIC activity data have also been used to illustrate concepts in public health calls to action in CDC reports: on CDIs (75% of cases had infection occurring outside of hospitals) (*31*), on CREs (92% of CRE episodes occurred in patients with health-care exposures) (*32*), and on the public health problem of incorrect inpatient antimicrobial drug use (37% of antimicrobial drug prescribing in selected scenarios could be improved) (*16*). In addition, data from candidemia surveillance were used in the World Health Organization’s first global report on antimicrobial resistance (*33*).

## Future of the EIP HAIC Activity

The accomplishments of the EIP HAIC activity have been numerous over a relatively short period of time, including delivery of data that have affected federal policy, programs, and operational approaches of HAI surveillance and prevention. However, as the landscape of HAI and antimicrobial drug–resistant infection prevention changes, the HAIC activity must constantly reassess priorities and direction. Reporting requirements related to HAIs as part of the Centers for Medicare and Medicaid Services quality reporting programs have expanded in recent years; data from the HAI and antimicrobial drug use prevalence survey show that ≈28% of all acute care hospital–related HAIs are now part of the Hospital Inpatient Quality Reporting Program of the Centers for Medicare and Medicaid Services (http://www.cms.gov/Medicare/Quality-Initiatives-Patient-Assessment-Instruments/HospitalQualityInits/HospitalRHQDAPU.html). The flexibility of the HAIC activity makes it well suited to fill gaps in facility-specific reporting as part of those programs, to contribute data on hospital HAIs not included in reporting programs, and to provide data on the large proportion of infections caused by health care–associated pathogens that occur outside acute care hospital settings. For example, as reporting to NHSN becomes increasingly robust for particular hospital-onset infections, the HAIC activity can adapt its surveillance approach to focus on cases in nonacute care or community settings, locations where high quality data would otherwise be lacking. Thus, the HAIC activity can provide an infrastructure that enables evaluation of progress of prevention efforts.

As reporting requirements become part of nonacute care settings, including long-term care or ambulatory care, the EIP HAIC activity will be well positioned to help determine selection of the highest-priority infection metrics in those settings. Periodic assessment of the spectrum of HAIs through time-limited activities, such as the point-prevalence surveys, will help CDC reassess priority infections for prevention efforts and determine needed modifications to reporting requirements for various types of health care facilities. Over the next decade, the HAIC activity can serve to identify new and emerging challenges involving HAIs occurring across the spectrum of health care delivery.

The HAIC activity can also continue to develop new techniques and respond to emerging and urgent issues related to HAI surveillance and antimicrobial resistance. With knowledgeable, state-based staff and existing networks in health care facilities and clinical microbiology laboratories, the HAIC activity can explore novel approaches to HAI tracking, accommodate shifting case definitions and approaches to defining antimicrobial resistance, and contribute valuable data to inform development and implementation of optimal definitions through ongoing collection and study of isolates linked to well-defined cases of infection.

Besides these functions, the HAIC activity provides an infrastructure for evaluating approaches to the prevention of HAIs and the spread of antimicrobial resistance by building on research and innovations tested and refined in smaller-scale or academic settings. The activity’s surveillance projects have firmly established outcome metrics and can therefore measure patient-centered outcomes after early adoption of new standards in HAI prevention efforts in acute or long-term care settings (e.g., the effects of hospital-based programs to reduce CDI in postdischarge settings). One challenge facing the HAIC activity in implementing these evaluations is the population-based nature of many of its surveillance projects. Currently, cases are defined in part on the basis of residency in the designated catchment area, and new approaches to enable capture of nonresident cases will be needed, particularly for work focused in acute care hospitals.

During the past 5 years, the HAIC activity infrastructure has adapted quickly to new challenges, additional pathogens, and new methods to accomplish its mission. Given the scope of the antimicrobial resistance problem and the aggressive timeline laid out in the US President’s September 2014 Executive Order (https://www.whitehouse.gov/the-press-office/2014/09/18/executive-order-combating-antibiotic-resistant-bacteria), the pace of work will need to be accelerated to make progress in reaching the targets outlined in the National Strategy for Combating Antibiotic-Resistant Bacteria (*34*). These targets include large reductions in incidence of multidrug-resistant *Pseudomonas aeruginosa*, invasive MRSA, CDI, and CRE. Through the EIP HAIC activity, CDC will be better and more rapidly able to identify populations at risk for antimicrobial drug–resistant infections associated with health care settings, to evaluate and refine prevention approaches, and to define critical links between disease severity or prevention and microbe characteristics. Furthermore, this program will serve as one of several that will assess the success of various components of the National Strategy towards achieving targets and providing data to empower public health and health care sectors to make progress toward eliminating HAIs.
